# Revitalizing the infection prevention workforce with a fellowship program for underrepresented groups

**DOI:** 10.1017/ice.2023.38

**Published:** 2023-12

**Authors:** Alison L. Nelson, Jacqueline Steiner, Diane Hanley, Tamar F. Barlam, Mari-Lynn Drainoni, Shana A. B. Burrowes, Cassandra M. Pierre

**Affiliations:** 1 Boston Medical Center, Boston, Massachusetts; 2 Section of Infectious Diseases, Department of Medicine, Boston University Chobanian & Avedisian School of Medicine, Boston, Massachusetts; 3 Department of Health Law, Policy & Management, Boston University School of Public Health, Boston, Massachusetts

## Abstract

Infection preventionist (IP) positions are difficult to fill, and future workforce shortages are anticipated. The IP field has less racial and ethnic diversity than the general nursing workforce or patient population. A targeted fellowship program for underrepresented groups allowed the recruitment and training of IPs while avoiding staffing shortages.

Infection preventionists (IPs) play an essential role in acute-care hospitals and other healthcare settings, but staffing these positions is challenging. A survey conducted by the Association for Professionals in Infection Control and Epidemiology (APIC) in 2015 found that >40% of the IP workforce was aged >55, and therefore could be anticipated to retire over the next decade.^1^ Another survey indicated that 25% of programs report at least 1 vacant position, with many remaining unfilled for extended durations.^2^ Recruitment of new IPs from nonnursing backgrounds, such as public health and microbiology, has been proposed^3^ but may not be sufficient to meet the need. The COVID-19 pandemic has placed additional strain on the field, contributing to burnout while increasing need for IP expertise. It is crucial not only to fill existing positions but also to expand the IP workforce to meet growing demand and responsibilities. Together with this challenge, it is also important to ensure that this field reflects the diverse patient population and healthcare workforce it serves.

Historically, nursing has had lower representation of structurally marginalized racial and ethnic groups compared to the general population. Increased hiring of Black and Hispanic or Latinx nurses has been observed in recent years^4^; however, pervasive gaps in racial and ethnic representation remain within the IP workforce. Demographic distributions for APIC surveys in 2015 and 2020 were unchanged, with >83% of Ips identifying as non-Hispanic white.^1,5^ Diversification of the healthcare workforce is critical to promote equitable healthcare outcomes, improve culturally competent care, enhance innovation, and improve financial performance.^6^ We have described an innovative model for internal recruitment, training, and certification of a diverse cohort of new Ips that allowed our hospital to address vacancies and to grow a department whose members now better reflect the community they serve.

## Methods

### Intervention site

Boston Medical Center (BMC) is a 514-bed, tertiary-care, teaching hospital in an urban setting, and it is New England’s largest safety-net hospital. Many patients come from underserved and structurally minoritized groups, as reflected by racial and ethnic identification, housing insecurity, immigration status, and socioeconomic status. The BMC Department of Hospital Epidemiology and Infection Control (HEIC) supports the acute-care hospital and 24 affiliated ambulatory-care sites. In 2021, the demographics for patients treated at the hospital were 32% Black, 24% Hispanic or Latinx, and 25% white. Registered nurses employed by the hospital were 18% Black, 4% Hispanic or Latinx, and 71% white. During the past several years, nursing leadership has made increasing racial and ethnic diversity of the nursing workforce a focus, with strategic goals to hire underrepresented group candidates for at least 40% of new graduate positions and at least 30% of nursing leadership positions.

In 2020, the BMC HEIC was staffed with 4 practicing IPs, with 3 anticipated to leave their position within 2–3 years. After efforts to hire experienced IPs externally were unsuccessful, approval was granted for 3 nursing IP fellowship positions.

### Intervention description

Facing staffing shortages that were particularly urgent in the setting of the COVID-19 pandemic, the BMC Nursing Department aimed to stabilize the IP work force, meet additional capacity needs, and increase its diversity through the creation of an Infection Prevention Fellowship (IPF). The fellowship was targeted to experienced nurses who identified as underrepresented racial and ethnic groups interested in pursuing careers in infection prevention. Applicants were required to have a bachelor’s degree, with a master’s degree preferred. The fellowship provided a 12-month program of hands-on training following the structure of the APIC Professional Practice Roadmap for Novices,^7^ an outline for knowledge, skill, and abilities needed to sit for the exam conveying Certification in Infection Prevention and Control (CIC). Of the IPF applicants, 7 were selected for interviews. Of these, 3 individuals—1 Latinx and 2 Black nurses—were offered the fellowship. They had work experience in critical care, inpatient wards, and occupational health.

The IP fellows began in spring 2021 under supervision of senior IP staff mentors. Initial orientation included shadowing, learning the basics of infection prevention, and joining professional groups and associations, including APIC and the National Healthcare Safety Network (NHSN). In addition to their learning responsibilities, the fellows’ specifically designated tasks included healthcare-associated infection (HAI) surveillance and reporting, outbreak investigations, task-group participation related to HEIC initiatives, and coordination with occupational health, as well as other domains identified in the Fellowship Roadmap.

The prior work experience and connections of the IP fellows allowed for their rapid incorporation into rounding in clinical spaces and real-time reinforcement of optimal infection control practices. Their presence also enabled HEIC to institute an initiative focused on review of central lines and indwelling urinary catheters and direct outreach to providers regarding possible device removal. Over the first year, the fellows reviewed ∼120 active devices per day and initiated queries regarding continued need on ∼5% of these, many of which were able to be removed within 1–2 days.

### Outcomes

As of fall 2022, all 3 IP fellows had completed IPF training. Presently, 2 have passed the CIC exam and the third fellow has scheduled an exam date for spring 2023. All 3 remain employed at BMC within HEIC. Following the success of the IPF program in achieving training goals and addressing existing vacancies, a fourth fellowship position was posted and filled in 2022.

## Discussion

To reinvigorate our IP workforce, our institution was able to recruit a diverse group of talented and well-respected individuals from within the nursing staff and provide them with an opportunity for career advancement. Their existing relationships, knowledge of nursing practices, and familiarity with the culture of the hospital allowed them to efficiently become trusted resources for both the general nursing staff as well as other members of the clinical care teams.

Infection preventionists provide an essential function in all healthcare environments, but maintaining appropriate staffing has been difficult at most healthcare facilities.^2^ Several strategies to bridge this gap have been suggested, including recruitment of individuals with degrees in nonnursing fields, such as public health professionals^3,8^ and medical technologists.^3,9^ However, it is likely that many IPs will continue to have a nursing background. Internal nursing staff is an ideal pool from which to draw qualified candidates from underrepresented groups. They are clinically experienced healthcare professionals. Additionally, nurses from underrepresented groups have equal or higher rates of attaining bachelor’s or master’s level nursing degrees compared to white nurses.^10^ They have been underutilized as a resource for recruitment for IP positions.

Reducing racial and ethnic disparities in the recruitment of healthcare personnel contributes to the improvement of culturally competent care, patient communication, patient health outcomes, and innovation.^6^ Diversity-focused recruitment within our workforce has resulted in the identification of individuals who were uniquely qualified to be leaders in our institution due to years of established relationships and trust. The nurses who participated in our IP fellowship are viewed as approachable and accessible by our healthcare workers for infection prevention–related questions and are well positioned to communicate staff concerns to HEIC leadership.

In summary, internal recruiting of a diverse panel of experienced nurses into a mentored IP fellowship program allowed our hospital to avoid anticipated staffing shortages, improve communication and outreach to staff, and ensure diverse perspectives are represented in our current and future efforts in infection control.
